# The crucial role of miR-126 on suppressing progression of esophageal cancer by targeting VEGF-A

**DOI:** 10.1186/s11658-016-0004-2

**Published:** 2016-07-28

**Authors:** Ranran Kong, Yuefeng Ma, Jie Feng, Shaomin Li, Wei Zhang, Jiantao Jiang, Jin Zhang, Zhe Qiao, Xiaoping Yang, Bin Zhou

**Affiliations:** 1 Department of Thoracic Surgery, The Second Affiliated Hospital of Xi’an Jiaotong University, 710004 Xi’an, Shaanxi China; 2 Department of Nephrology, The First Affiliated Hospital, Xi’an Jiaotong University, 710061 Xi’an, Shaanxi China

**Keywords:** miR-126, Esophageal cancer, VEGF-A, Tumorgenesis, Cell proliferation, Lentivirus package, MTT assay, Xenograft model

## Abstract

**Background:**

miR-126 is a key regulator of oncogenic processes. It is functionally linked to cellular proliferation, survival and migration. Vascular endothelial growth factor A (VEGF-A), which is regarded as a tumorgenesis activator, could directly target miR-126 in several tumors. However, the mechanism in esophageal cancer remains unclear.

**Methods and results:**

In this study, the expression of miR-126 and VEGF-A were assessed in esophageal cancer tissues and esophageal cancer cell lines. We found that miR-126 has significantly lower expression in esophageal cancer tissues and esophageal cancer cell lines than in healthy tissues, while the expression of VEGF-A is high. Luciferase reporter assays were performed to investigate the relationship between VEGF-A and miR-126. We confirmed that VEGF-A is a target for miR-126. Furthermore, the proliferation of esophageal cancer cells with miR-126 overexpression and miR-126 knockdown was monitored using the MTT assay. The results showed that miR-126 could inhibit esophageal cancer cell proliferation in vitro. The effect of miR-126 was also detected in BALB/c nude mice with transplanted esophageal cancer cells. In vivo study showed that tumor growth was significantly suppressed by miR-126 overexpression.

**Conclusions:**

We believe that restoring miR-126 levels may be a promising therapeutic approach in cases of esophageal cancer.

## Introduction

Over the past few decades, esophageal cancer incidence and mortality increased worldwide. Previous studies showed that microRNA (miRNA) expression and esophageal cancer are closely related. miR-203 and miR-205 expression in esophageal squamous cell carcinoma is significantly lower than in normal epithelial tissues, while the expression of miR-21 is significantly higher than in the normal epithelial tissues [1].

miRNA-126 (miR-126), an endothelial cell-restricted miRNA, was found to regulate developmental angiogenesis [2]. miR-126 is located within intron 7 of epidermal growth factor-like domain 7 (EGFL7). It acts as a tumor suppressor gene in many malignant tumor cells, inhibiting the progression of some cancers via negative control of proliferation, migration, invasionand cell survival. However, in some cases, miR-126 supports cancer progression via promotion of blood vessel formation [3].

VEGF can promote endothelial cell proliferation and play significant roles in angiogenesis and tumor growth. During tumor expansion, VEGF can increase the permeabilization of blood vessels and the production of new blood vessels [4]. VEGF-A is a target gene of miR-126. Downregulation of miR-126 increases VEGF-A activity in lung cancer [5] and breast cancer [6].

Although studies on miRNA in esophageal cancer have been reported, the underlying mechanisms have not been fully elucidated. Therefore, this study focused on the regulatory functions of miRNA-126 in the progression of esophageal cancer.

## Materials and methods

### Cell cultures and animal subjects

The human esophageal adenocarcinoma cell lines OE19 and OE33 were purchased from the European Collection of Cell Cultures. The 293T (ATCC CRL-3216) cell line and JH-EsoAd1 cells were purchased from the American Type Culture Collection.

Cells were cultured in RPMI 1640 medium (R0883; Sigma) supplemented with 2 mM glutamine, 10 % fetal bovine serum (FBS), 100 U/ml penicillin G and 100 mg/ml streptomycin (Imperial Laboratories) and maintained in monolayer culture at 37 °C in an incubator of humidified air with 5 % CO_2_.

Male BALB/c nude mice aged 8–10 weeks were obtained from the Animal House of Xi’an Jiaotong University and their conditions were approved by the Animal Care and Use Committee of the Second Affiliated Xi’an Jiaotong University.

### Tissue sample collection

A set of 13 esophageal cancer tissue samples and paired adjacent normal tissue samples were obtained from patients undergoing esophageal cancer surgery at the Second Affiliated Hospital of Xi’an Jiaotong University. All of the esophageal cancer cases were pathologically confirmed by two independent experts. The collected tissues were frozen at −80 °C for further analysis. Informed consent was obtained, and this study was in accordance with the Declaration of Helsinki and approved by the Human Ethics Committee of the Second Affiliated Hospital of Xi’an Jiaotong University.

### RNA isolation and quantitative RT-PCR

RNA was extracted from the 13 human esophageal cancer tissues and cultured cells using Trizol Reagent (Invitrogen) according to the manufacturer’s instructions. It was then reverse transcribed into cDNA using aPrimeScript RT Reagent Kit (TaKaRa). The cDNA underwent PCR with an TaKaRaBio SYBR Green Master Mix kit using the miR-126 primer set as follows: sense, 5’-CAA CAG AAG GGG CAG GTT GCC CGG AGC-3’; and antisense, 5’-ATT CTG ATC ACG CCT AAG TAC GTC GGG GC-3’. The U6 primers set was: sense, 5’-ATC CGC AAA GAC CTG T-3’; and antisense, 5’-GGG TGT AAC ACT AAG-3’ (Sangon). The relative levels of miR-126 transcripts were normalized to the control U6 mRNA. The cycle number at which the reaction crossed an arbitrarily placed threshold (C_t_) was determined for each gene, and the relative amount of each miRNA to U6 snRNA was calculated using the equation 2^-ΔΔ*C*t^, where ΔΔC_t_ = (*C*_tmiRNA_ - *C*_tU6 snRNA_) _transfected_ - (*C*_tmiRNA_ - *C*_tU6 snRNA_)_control_. Each experiment was performed in triplicate.

### Plasmid construction and lentivirus package

The lentiviral vector pCDH-CMV (pLV, System Biosciences, SBI) was used to construct the pLV-mir-126 plasmid. The template was the human genome DNA of miR-126 (Accession number: NCBI Reference Sequence: NR_029695.1). Lentiviral constructs contained miR-126 (LV-miR-126) and anti-miR-126 (LV-anti-miR-126) along with the miR negative control (LV-miR-NC). To produce the lentivirus, three plasmid DNAs were individually transfected into 29 cells using psPAX2, a pMD2G packaging construct, and lipofectamin plus reagent (Invitrogen) according to the manufacturer’s instructions. After 8 h, the original medium was replaced with fresh medium, and the lentiviral supernatant was collected 48 h later. The expression level of green fluorescent protein (GFP) was measured to establish the titer of the virus, according to the manufacturer’s instruction.

### Luciferase reporter assay

293 cells were seeded in 96-well plates and grown in RPMI 1640 containing 10 % FBS at 37 °C in a humidified atmosphere containing 5 % CO_2_. After 24 h, luciferase reporter plasmids were transfected separately or co-transfected with VEGF-A wild-type 3’-UTR and VEGF-A mutation-type 3’-UTR. Firefly and renilla luciferase activities were measured 48 h after transfection using the Dual-Glo Luciferase Assay System (Promega). Firefly luciferase was normalized to renilla luciferase activity.

### In vivo tumor xenograft model

1 × 10^7^ cells stably transfected with LV-miR-126, LV-anti-miR-126 and lenti-miR-NC were injected subcutaneously into the right foreleg armpit of three groups of 18–26 g male BALB/c nude mice (8 mice/group) to establish a cancer model. The tumor size was detected every week using a caliper. At 42 days after inoculation, all of the mice were killed and the tumor masses were successively excised, weighed, photographed and subjected to western blot and luciferase reporter assays.

### Western blotting analysis

Immunoblotting was performed to detect the expression of VEGF-A in the cancer tissues and in the human esophageal adenocarcinoma cell lines OE33 and JH-EsoAd1, which had been infected with the recombinant lentivirus. For western blot analysis, the samples were lysed using the RIPA buffer (Pierce) in the presence of a protease inhibitor cocktail (Pierce). Samples with 25 μg of total protein were resolved by SDS-PAGE on a 10 % gel and transferred onto an NC membrane. The blot was incubated with TBST with 1:500 goat primary antibodies against human VEGF-A (sc-152), followed the secondary HRP-conjugated anti-goat antibody (both from Santa-Cruz Biotechnology). After washing, the bands were detected using chemiluminescence (ECL detection kit) and imaged with Kodak film. Glyceraldehyde-3-phosphate dehydrogenase (GAPDH) was used as an endogenous protein for normalization.

### MTT assay

Cell proliferation was analyzed using the3-(4,5-dimethylthiazol-2-yl)-2,5-diphenyltetrazoliumbromide (MTT) assay. Cells were seeded into 96-well plates (5 × 10^3^ cells/well) directly or at 1 day after stable transfection, and incubated for 1, 3,5, 7, 9 and 11 days. After incubation with 25 μl of MTT (5 mg/ml, Sigma) at 37 °C for 4 h, the supernatants were removed, and 150 μl of dimethylsulfoxide (DMSO, Sigma) was added to each well. The absorbance value (OD) of each well was measured at 490 nm. All experiments were performed three times and the average results were calculated.

### Statistical analysis

Results were analyzed statistically using Student’s *t-*test for comparisons between two groups. Data are presented as the means ± SD. Correlation parameters were submitted to Pearson and non-parametric Spearman correlations. A *P* value less than 0.05 was considered to indicate statistical significance. Analyses were performed using SPSS 17.0 for Windows (SPSS).

## Result

### Expression of miR-126 and VEGF-A in esophageal cancer tissue samples and cells

**Fig. 1:**
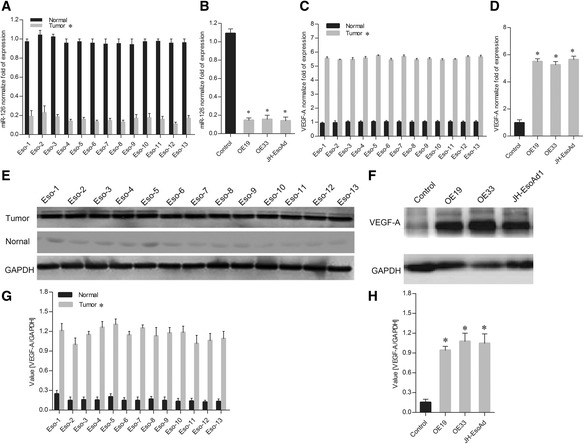
Expression of miR-126 and VEGF-A in esophageal cancer tissues and esophageal carcinoma cell lines. **a** qRT-PCR analysis of miR-126 expression in 13 esophageal cancer tissues and paired adjacent normal tissues. **b** qRT-PCR analysis of miR-126 expression in three esophageal carcinoma cell lines and a control cell line. **c** qRT-PCR analysis of VEGF-A expression in the esophageal cancer tissues and paired adjacent normal tissues. **d** qRT-PCR analysis of VEGF-A expression in three esophageal carcinoma cell lines and a control cell line. **e** Western blot analysis of VEGF-A expression in esophageal cancer tissues and paired adjacent normal tissues. **f** Western blot analysis of VEGF-A expression in three esophageal carcinoma cell lines and a control cell line. **g** Quantitation of protein expression for Fig. 1e. **h** Quantitation of protein expression for Fig. 1f. The data is shown as the means ± SD. *p <0.01, compared to the control group

The expression levels of miR-126 in 13 esophageal cancer tissue samples and paired adjacent normal tissue samples were determined using quantitative RT-PCR. The results showed that the expression level of miR-126 was approximately 7 times lowerin all 13 esophageal cancer tissue samples than that in the normal tissues (Fig. 1a).

Furthermore, we also tested the expression of miR-126 in the OE19, OE33 and JH-EsoAd1 esophageal carcinoma cell lines. Compared with the control cell lines, the expression levels of miR-126 in the esophageal carcinoma cell lines were also significantly lower (Fig. 1b). This indicts that miR-126 might play a negative role in esophageal carcinogenesis.

The expression of VEGF-A in all 13 cancer and normal tissue samples and the carcinoma cell lines was also assessed using qRT-PCR and western blot. Compared with the control, the VEGF-A expression levels were significant upregulated (Fig. 1) at both the mRNA and protein levels.

### Inhibition of VEGF-A expression in esophageal cancer cells with miR-126 overexpression

**Fig. 2:**
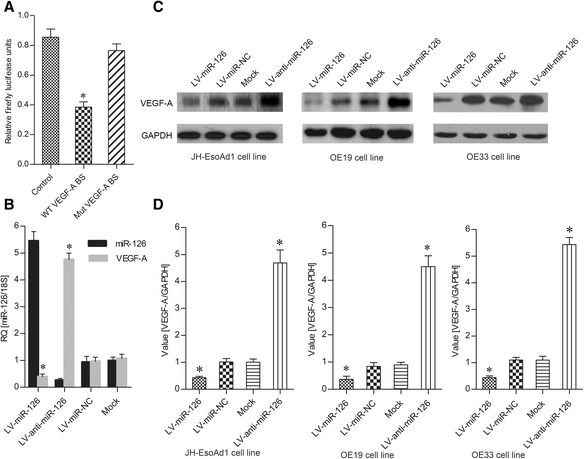
The expression of VEGF-A is regulated by miR-126. **a** Relative firefly luciferase units in 293 cells transfected with WT VEGF-A or Mut VEGF-A. **b** Expression of miR-126 and VEGF-A in 293 cells treated with different vectors. **c** Western blot analysis of VEGF-A expression in three esophageal carcinoma cell lines treated with different vectors. **d** Quantitation of protein expression for Fig. 2c. The data is shown as the means ± SD. *p <0.01, compared to the controls LV-miR-NC and Mock

To find direct targets of miR-126, luciferase activity was measured by the co-transfection of the VEGF-A wild-type 3’-UTR with miR-126. The miRNA decreased luciferase activity, whereas this effect was completely ablated by deletion of the miR-126-binding site in the VEGF-A 3’-UTR (Fig. 2a). These results suggest that VEGF-A was the direct target of miR-126.

To investigate the regulatory role of miR-126 on VEGF-A expression in esophageal cancer in vitro, VEGF-A expression was detected in esophageal cancer cell lines with miR-126 overexpression and knockdown. Compared to the lentivirus miR negative control (LV-miR-NC) and the vector without lentivirus (Mock), the expression of miR-126 in cells infected with recombinant lentivirus miR-126 was significantly increased, whereas with the treatment with anti-miR-126 inhibitor (LV-anti-miR-126), the miR-126 expression decreased significantly (Fig. 2b). Furthermore, the overexpression and inhibition of miR-126 in esophageal cancer cells could downregulate or upregulate the expression of VEGF-A both at the mRNA and protein levels (Fig. 2b–d). These results demonstrate that overexpression of miR-126 could inhibit VEGF-A expression.

### miR-126 inhibits cell proliferation in esophageal cancer

**Fig. 3:**
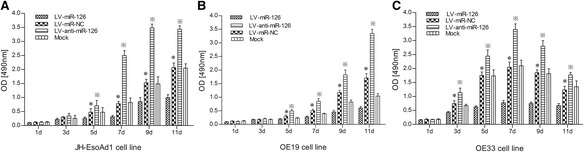
The proliferation of esophageal cancer cells as determined using the MTT assay. **a** Effects of LV-miR-126 on the cell line JH-EsoAd1. **b** Effects of LV-miR-126 on OE19. **c** Effects of LV-miR-126 on OE33. The data is shown as the means ± SD. *p <0.01, compared to LV-miR-NC and Mock

The MTT assay was employed to investigate the effects of miR-126 on cell proliferation in esophageal cancer. LV-miR-126 was transfected into esophageal cancer cells for this assay. The results revealed that exogenous expression of miR-126 could inhibit the proliferation of JH-EsoAd1, OE19 and OE33 cells (Fig. 3). Statistical analysis suggested similar effects of miR-126 on cell proliferation*.* These results suggest that miR-126 could inhibit VEGF-A expression and then inhibit esophageal cancer cell proliferation in vitro*.*

### Effect of miR-126 expression on the progression of esophageal cancer in vivo

**Fig. 4:**
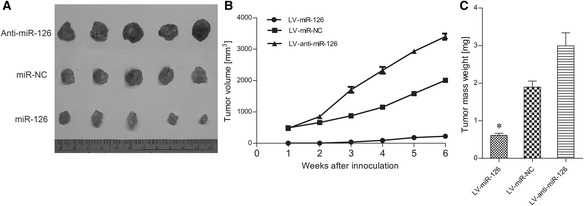
The effects of miR-126 on tumor growth in nude mice inoculated with esophageal cancer cells. **a** The measurement of tumor volumes in each group. **b** The tumor volumes over the experimental period. **c** The tumor mass weights for each group. The data is shown as the means ± SD. *p <0.01, compared to LV-miR-NC and Mock

To figure out whether changes in miR-126 expression could influence the growth of tumorsin vivo, three groups of nude mice were inoculated with OE33 cells that had been stably transfected with recombinant lentivirus miR-126 (LV-miR-126), anti-miR-126 inhibitor (LV-anti-miR-126) and lentivirus miR negative control (LV-miR-NC). Tumor formation was observed and tumor weight was measured in these three groups (Fig. 4). These results showed that the ectopic expression of miR-126 inhibited tumorigenesis in vivo. The average tumor weight of mice inoculated with lenti-miR-126-transfected OE33 cells was significantly lower on day 42 (*p* < 0.01) than that for mice inoculated with anti-miR-126 transfected OE33 cells and the miR-NC negative control group. These results revealed that miR-126 could inhibit esophageal cancer growth by downregulating VEGF-A expression.

## Discussion

It has been demonstrated that abnormal expression of miR-126 correlates with human tumorigenesis and that miR-126 has roles in cancers of the gastrointestinal tract, genital tracts, breasts, thyroid, lungs and some other tissues and organs [7]. Li found that miR-126 expression was downregulated in gastric carcinoma tissues compared with matched non-cancer tissues when assessed with qRT-PCR [8]. In addition, miR-126 was identified as a tumor suppressor in gastric cancer and as an inhibitor of V-crk avian sarcoma virus CT10 oncogene homolog-like (CRKL)thanks to its targeting of the 3’ UTR region of CRKL mRNA [9] or VEGF-A mRNA [10]. It has also been determined that miR-126 expression is deregulated in colorectal cancer [11–14]. For the mechanism of its repression of colon cancer proliferation and invasion, Li reported that miR-126 negatively regulates the expression of CXCR4 and inhibits the RhoA/ROCK (Rho-associated proteinkinase) signalling pathway [15]. In oral cancer, Sasahira has reported that low miR-126 expression is associated with tumour progression due to upregulation of the VEGF-A signal [16]. In lung carcinoma, miR-126 expression was found to be reduced [17].

Crawford discovered that miR-126 can suppress lung cancer invasion by directly targeting CRK [18], and Liu reported that miR-126 suppresses the expression of VEGF-A, inhibiting cancer cell growth [19]. Zhu suggests that enhanced expression of miR-126 elevates the sensitivity of non-small cell lung cancer cells to anticancer therapy via negative regulation of the VEGF/PI3K/Akt/MRP1 signalling pathway [20]. These studies suggest that miR-126 is a potential tumor suppressor gene. Based on these reports, we postulate that miR-126 plays a critical but as-yet-unknown role in human esophageal cancer.

Recently, Koumangoye et al. reported that miR-31 is significantly decreased in esophageal cancer cells, while upregulation of miR-31 inhibits growth, migration and invasion of esophageal adenocarcinoma (EAC) and squamous cell carcinoma (ESCC) cell lines [21]. Yu et al. suggested that miR-130b plays an oncogenic role by repressing PTEN expression in esophageal squamous cell carcinoma cells [22].

Liu et al. discovered that microRNA126-3p (miR-126) was significantly downregulated in esophageal squamous cell carcinoma (ESCC), and its downregulation correlated with poor ESCC prognosis. Furthermore, they found downregulation of miR-126 was due to promoter hypermethylation of its host gene, Egfl7 [23]. In another study, Liu et al. reported that when miR-126 in the esophageal cancer tissues was compared with its levels in matched normal tissues using miRNA microarrays, it was found to be reduced [24].

However, the effect of miR-126 in esophageal cancer remains unclear. In this study, we showed that miR-126 was bound to VEGF-A 3’-UTR, which resulted in reduced expression of VEGF-A in esophageal cancer cells, which inhibited esophageal cancer growth. We found miR-126 had lower expression in esophageal cancer samples compared to adjacent normal samples around the cancer, which is consistent with earlier results [25].

However, there were some controversies for miR-126 in esophageal cancer. Hu discovered that miRNA-126 failed to show a relationship to the outcome of patients with esophageal adenocarcinoma [26]. These contradictory results might due to the distinct biology and clinical features of esophageal squamous carcinoma in our study and esophageal adenocarcinoma in the previous study. The differences in the subject populations (Asian vs. American) might also contribute to this.

Furthermore, in ourqRT-PCR and western blot assays, we discovered that the VEGF-A expression levels were upregulated in human esophageal cancer cells and negatively correlated with miR-126. These results suggest that miR-126 as a possible negative regulatory role in VEGF-A expression. It has been demonstrated that downregulated miR-126 increases VEGF-A activity in oral cancer, lung cancer and breast cancer [16, 19, 27] and that miR-126 may act as a tumor suppressor by regulating VEGF-A expression in esophageal cancer.

To understand the molecular mechanisms of miR-126 suppressing esophageal cancer cells, we searched for putative miR-126 targets. VEGF-A is an important invasion and metastasis factor [28, 29]. This has led to the hypothesis that VEGF-A is atarget for miR-126. Based on luciferase activity assays, we found that VEGF-A is a direct target of miR-126and confirmed that miR-126 negatively regulates VEGF-A expression in overexpressed miR-126 esophageal cancer cells from the lines JH-EsoAd1, OE19 and OE33. To further clarify this, the MTT assay was carried out, and the results showed that miR-126 could inhibit esophageal cancer cells proliferation in vitro. More importantly, ectopic expression of miR-126 in nude mouse inhibited tumorigenesis.

Our result was consistent with a previous study, which demonstrated that downregulation of miR-126 was reverse correlated with VEGF-A expression in gastric cancer. Furthermore, Chen et al. also suggested that the downregulation of miR-126 inhibited gastric tumor growth and tumor angiogenesis through activation ofAkt, mTORand Erk1/2 of VEGF-A signaling downstream genes [9, 30].

In conclusion, miR-126 was naturally complementary to the VEGF-A 3’-UTR and could downregulate overexpression of VEGF-A in esophageal cancer cells, thus inhibiting the esophageal cancer growth.

## Abbreviations

Akt: Protein kinase B
CRKL: V-crk avian sarcoma virus CT10 oncogene homolog-like
CXCR4: c-x-c chemokine receptor type 4
EGFL7: Epidermal growth factor-like domain 7
Erk1/2: Extracellular regulated protein kinases 1/2
ESCC: Esophageal squamous cell carcinoma
GAPDH: Glyceraldehyde-3-phosphate dehydrogenase
GFP: Green fluorescent protein
HRP: Horse reddish peroxidase
LV-miR-126: Lentiviral vector-miR-126
LV-miR-NC: Lentiviral vector-miR- negative control
miR-126: microRNA 126
miRNA: microRNA
MRP1: Multi-drug resistant associate protein
mTOR: Mechanistic Target of Rapamycin
MTT: 3-(4,5-dimethyl-2-thiazolyl)-2,5-diphenyl-2-H-tetrazolium bromide
OD: Absorbance value
PI3K: Phosphatidylinositol 3 kinase
RhoA: Ras homolog gene family member A
ROCK: Rho-associated protein kinase
VEGF-A: Vascular endothelial growth factor A
